# Clinical markers of disease progression in the prodromal to overt alpha-synucleinopathy continuum

**DOI:** 10.1093/brain/awag193

**Published:** 2026-05-29

**Authors:** Monica Roascio, Elena Antelmi, Luca Baldelli, Francesco Biscarini, Enrica Bonanni, Valerio Brunetti, Elena Capriglia, Claudio Geromino, Francesca Casoni, Flavia Cirillo, Giacomo Della Marca, Felice Di Laudo, Luigi Ferini-Strambi, Mariana Fernandes, Michela Figorilli, Andrea Galbiati, Gian Luigi Gigli, Biancamaria Guarnieri, Domeniko Hoxhaj, Giuseppe Lanza, Claudio Liguori, Michelangelo Maestri Tassoni, Silvia Maio, Gaetano Malomo, Sara Marelli, Pietro Mattioli, Martina Mulas, Beatrice Orso, Matteo Pardini, Giuseppe Plazzi, Federica Provini, Gaia Pellitteri, Monica Puligheddu, Michele Terzaghi, Amélie Pelletier, Ronald B Postuma, Gabriele Arnulfo, Dario Arnaldi

**Affiliations:** Department of Informatics, Bioengineering, Robotics and System Engineering (DIBRIS), University of Genoa, Genoa 16145, Italy; Department of Engineering for Innovative Medicine, University of Verona, Verona 37134, Italy; Department of Biomedical and Neuromotor Sciences, University of Bologna, Bologna 40138, Italy; UOC Clinica Neurologica Rete Metropolitana (NEUROMET), IRCCS Istituto delle Scienze Neurologiche di Bologna, Bologna 40139, Italy; Department of Biomedical and Neuromotor Sciences, University of Bologna, Bologna 40138, Italy; UOC Clinica Neurologica, IRCCS Istituto delle Scienze Neurologiche di Bologna, Bologna 40139, Italy; Neurology Unit, Department of Clinical and Experimental Medicine, University of Pisa, Pisa 56126, Italy; UOC di Neurologia, Dipartimento di Neuroscienze, Organi di Senso e Torace, Fondazione Policlinico Universitario Agostino Gemelli IRCCS, Rome 00168, Italy; Department of Neurosciences, Università Cattolica del Sacro Cuore, Rome 00168, Italy; Department of Brain and Behavioral Sciences, University of Pavia, Pavia 27100, Italy; Interdepartmental Sleep Medicine Centre, University of Cagliari, Cagliari 09124, Italy; Neurology Unit, Azienda Ospedaliero-Universitaria, Cagliari 09042, Italy; Sleep Disorders Centre, Department of Clinical Neurosciences, IRCCS San Raffaele Hospital, Milan 20127, Italy; Neurology Unit, University Hospital of Rome Tor Vergata, Rome 00133, Italy; UOC di Neurologia, Dipartimento di Neuroscienze, Organi di Senso e Torace, Fondazione Policlinico Universitario Agostino Gemelli IRCCS, Rome 00168, Italy; Department of Neurosciences, Università Cattolica del Sacro Cuore, Rome 00168, Italy; Department of Biomedical and Neuromotor Sciences, University of Bologna, Bologna 40138, Italy; Division of Neuroscience, IRCCS San Raffaele Hospital and Vita-Salute San Raffaele University, Milan 20132, Italy; Department of Systems Medicine, University of Rome Tor Vergata, Rome 00133, Italy; Interdepartmental Sleep Medicine Centre, University of Cagliari, Cagliari 09124, Italy; Neurology Unit, Azienda Ospedaliero-Universitaria, Cagliari 09042, Italy; Division of Neuroscience, IRCCS San Raffaele Hospital and Vita-Salute San Raffaele University, Milan 20132, Italy; Neurology, Department of Medicine (DMED), University of Udine, Udine 33100, Italy; Center of Sleep Medicine, Villa Serena Hospital, Città S. Angelo, Pescara 65013, Italy; Fondazione Villaserena per la Ricerca, Città S. Angelo, Pescara 65013, Italy; Neurology Unit, Department of Clinical and Experimental Medicine, University of Pisa, Pisa 56126, Italy; Clinical Neurophysiology Research Unit and Sleep Research Centre, Oasi Research Institute-IRCCS, Troina 94018, Italy; Department of Surgery and Medical-Surgical Specialties, University of Catania, Catania 95123, Italy; Neurology Unit, University Hospital of Rome Tor Vergata, Rome 00133, Italy; Department of Systems Medicine, University of Rome Tor Vergata, Rome 00133, Italy; Neurology Unit, IRCCS Neuromed, Pozzilli (Isernia) 86077, Italy; Neurology Unit, Department of Clinical and Experimental Medicine, University of Pisa, Pisa 56126, Italy; Department of Systems Medicine, University of Rome Tor Vergata, Rome 00133, Italy; Department of Brain and Behavioral Sciences, University of Pavia, Pavia 27100, Italy; Division of Neuroscience, IRCCS San Raffaele Hospital and Vita-Salute San Raffaele University, Milan 20132, Italy; Sleep Disorders Centre, Neurology, IRCCS San Raffaele Hospital, Milan 20127, Italy; Department of Neuroscience, Rehabilitation, Ophthalmology, Genetics, Maternal and Child Health (DINOGMI), Clinical Neurology, University of Genoa, Genoa 16132, Italy; Clinical Neurophysiology, IRCCS Azienda Ospedaliera Metropolitana, Ospedale Policlinico San Martino, Genoa 16132, Italy; Interdepartmental Sleep Medicine Centre, University of Cagliari, Cagliari 09124, Italy; Neurology Unit, Azienda Ospedaliero-Universitaria, Cagliari 09042, Italy; Department of Neuroscience, Rehabilitation, Ophthalmology, Genetics, Maternal and Child Health (DINOGMI), Clinical Neurology, University of Genoa, Genoa 16132, Italy; Department of Neuroscience, Rehabilitation, Ophthalmology, Genetics, Maternal and Child Health (DINOGMI), Clinical Neurology, University of Genoa, Genoa 16132, Italy; Clinical Neurology, IRCCS Azienda Ospedaliera Metropolitana, Ospedale Policlinico San Martino, Genoa 16132, Italy; UOC Clinica Neurologica, IRCCS Istituto delle Scienze Neurologiche di Bologna, Bologna 40139, Italy; Department of Biomedical, Metabolic and Neural Sciences, University of Modena and Reggio Emilia, Modena 41125, Italy; Department of Biomedical and Neuromotor Sciences, University of Bologna, Bologna 40138, Italy; UOC Clinica Neurologica Rete Metropolitana (NEUROMET), IRCCS Istituto delle Scienze Neurologiche di Bologna, Bologna 40139, Italy; Clinical Neurology Unit, Head-Neck and Neurosciences Department, Santa Maria Della Misericordia University Hospital, Udine 33100, Italy; Interdepartmental Sleep Medicine Centre, University of Cagliari, Cagliari 09124, Italy; Neurology Unit, Azienda Ospedaliero-Universitaria, Cagliari 09042, Italy; Department of Brain and Behavioral Sciences, University of Pavia, Pavia 27100, Italy; Sleep Medicine Centre, IRCCS Mondino Foundation, Pavia 27100, Italy; Centre for Advanced Research in Sleep Medicine, Hôpital du Sacré-Cœur de Montréal, Montréal, Quebec H4J 1C5, Canada; Department of Neurology, Research Institute of McGill University Health Centre, Montréal, Quebec H3H 2R9, Canada; Centre for Advanced Research in Sleep Medicine, Hôpital du Sacré-Cœur de Montréal, Montréal, Quebec H4J 1C5, Canada; Department of Neurology and Neurosurgery, Montréal Neurological Institute, McGill University, Montréal, Quebec H3A 2B4, Canada; Department of Informatics, Bioengineering, Robotics and System Engineering (DIBRIS), University of Genoa, Genoa 16145, Italy; Laboratorio di Medicina Computazionale e Tecnologica, IRCCS Istituto Giannina Gaslini, Genoa 16147, Italy; Department of Neuroscience, Rehabilitation, Ophthalmology, Genetics, Maternal and Child Health (DINOGMI), Clinical Neurology, University of Genoa, Genoa 16132, Italy; Clinical Neurophysiology, IRCCS Azienda Ospedaliera Metropolitana, Ospedale Policlinico San Martino, Genoa 16132, Italy

**Keywords:** idiopathic/isolated rapid-eye-movement sleep behaviour disorder, clinical disease progression, neurodegeneration biomarkers

## Abstract

Clinical progression from prodromal to overt stages of alpha-synucleinopathies is highly heterogeneous, and there is an urgent need for reliable clinical progression markers. Exploiting the Disease Course Mapping (DCM) model, we investigated how clinical signs evolve in patients with idiopathic/isolated REM sleep behaviour disorder (iRBD), extracting clinical progression measures for use at the single-subject level. Furthermore, we correlated them with both established and innovative neurodegeneration biomarkers.

We trained a DCM model using cognitive and motor scores of a longitudinal cohort of 766 patients with iRBD (166 female, 67.9 ± 7.4 years). We personalized the model by extracting three parameters to describe the single subject in comparison to the averaged population data. We tested the model on a blind set of 49 patients with iRBD (seven female, 68.5 ± 7.1 years) who underwent both longitudinal clinical evaluations and instrumental evaluation at the first observation. In the blind set, we correlated the individual model parameters with presynaptic dopaminergic impairment, an established biomarker of substantia nigra neurodegeneration, and cortical electrophysiological dysfunction—measured by high-density electroencephalography (HD-EEG)—an innovative neurodegeneration biomarker.

We identified three individual clinical markers reflecting early/late (time-shift, τ) and fast/slow (acceleration factor, α) disease progression, as well as the individual clinical trajectory (i.e. earlier motor or cognitive impairment, intermarker spacing, ω). The individual model parameters are significantly associated with phenoconversion, with a 73% chance of distinguishing between clinically stable patients (non-converters) and those converting during the longitudinal observation to an overt alpha-synucleinopathy (converters). Motor scores progress 35% faster than cognitive scores in our iRBD cohort. Converter iRBD patients exhibited a faster and earlier disease progression than non-converters, and on average, they showed an earlier worsening of motor scores than cognitive scores, regardless of the clinical diagnosis of overt parkinsonism. Patients with iRBD who developed parkinsonism worsened earlier than those who developed dementia. At baseline, an earlier progression was related to presynaptic dopaminergic impairment and higher phase synchronization in the theta band (4–8 Hz). Higher synchronization in the theta band was also associated with an earlier worsening of motor scores than cognitive scores.

In this study, we investigated a large longitudinal iRBD cohort, applying an advanced disease progression model. We found three individual clinical markers that were able to monitor disease progression and showed significant association with both established and innovative neurodegeneration biomarkers. We suggest that these clinical markers could be used as efficacy end points in disease-modifying clinical trials.

## Teaser

Roascio *et al.* trained a machine learning model to track clinical progression in the prodromal to overt alpha-synucleinopathies continuum. They identified three individual parameters, linked to cortical and subcortical neurodegeneration metrics, that could serve as reliable biomarkers of disease progression.

**See Nepozitek *et al*. (https://doi.org/10.1093/brain/awag306) for a scientific commentary on this article.**

## Introduction

Idiopathic/isolated REM sleep behaviour disorder (iRBD) is a parasomnia characterized by complex and often violent behaviours occurring during REM sleep due to the loss of the physiological muscle atonia.^1^ Idiopathic/isolated RBD is considered a prodromal stage of alpha-synucleinopathies; indeed, most patients with iRBD develop parkinsonism or dementia over time.^2^ However, patients with iRBD experience a range of symptoms and signs, with varying timing and manifestations, making it difficult to predict the phenoconversion into the overt alpha-synucleinopathies stage.^2^

The phenoconversion event is a fixed, binary outcome that is characterized by the fulfilment of clinical criteria for Parkinson’s disease (PD), dementia with Lewy bodies (DLB) or multiple system atrophy (MSA).^3-5^ However, the clinical features that are used in disease definitions do not occur acutely, but rather slowly, over a time frame of several years. Therefore, phenoconversion diagnosis oversimplifies a progressive transition from a clinical condition that mildly affects daily living activities (i.e. prodromal stages) to overt stages significantly impairing quality of life (i.e. overt parkinsonism and dementia). Thus, it is of paramount importance to identify clinical and instrumental measures that can efficiently monitor disease progression along the alpha-synucleinopathy continuum.^6-9^ In line with this concept, a recent study showed that the best progression marker from prodromal to overt stages in patients with iRBD is not the phenoconversion diagnosis, but rather a meaningful worsening of either motor or cognitive functions, as investigated by the Movement Disorder Society–Unified Parkinson’s Disease Rating Scale, motor section (MDS-UPDRS-III) and the Montreal Cognitive Assessment (MoCA) test.^6^ Any clinical measures of progression should be valid at the single-subject level to translate the identified metrics into clinical practice. A previous study used advanced standard statistical analysis to observe the progression of cognitive and motor scores.^7^ However, this type of analysis focuses on a single event (e.g. phenoconversion) and does not always consider the longitudinal nature of the data. It also presents challenges in managing missing data. In contrast, machine-learning models are not limited to a single event but capture the entire course of the disease. Furthermore, they can identify complex, non-linear relationships. The Disease Course Mapping (DCM) model is a recently developed non-linear mixed-effects statistical model able to estimate a unified spectrum of disease progression from a given population.^10^ The DCM model is mapped to individual data and provides metrics that compare individual progression with the progression of a selected population.

Beyond clinical monitoring of disease progression, a more comprehensive view of degeneration can be obtained by incorporating biomarkers. The most established neurodegeneration biomarker in alpha-synucleinopathies is presynaptic dopaminergic impairment, as measured by dopamine transporter single-photon emission tomography (DaT-SPECT).^11,12^ Indeed, the levels of DaT support the clinical identification of patients affected by overt PD or DLB in comparison with both healthy controls and with other diseases without nigro-striatal dopaminergic impairment, such as essential tremor and Alzheimer’s disease.^13^ Presynaptic dopaminergic damage is among the strongest risk factors for short-term phenoconversion in patients with iRBD.^14,15^

In addition, promising results have been obtained from the neurophysiological evaluation of global cortical synchronization and brain critical dynamics using high-density EEG (HD-EEG) recordings.^16-19^ The brain criticality hypothesis posits that the brain is a complex system that operates near a phase transition between hypersynchrony (supercritical) and asynchrony (subcritical) phases^20,21^ and can also show bistable dynamics.^22-26^ Physiological brain activity is characterized by an optimal excitation-inhibition (E/I) balance,^27^ intermediate levels of neuronal synchronization^28^ and mild bistability.^26^ In neurodegeneration, the working point appears to shift towards a hyperexcited and fully synchronized state,^17,29,30^ characterized by strong levels of bistability.^17^ Idiopathic/isolated RBD patients with high short-term phenoconversion likelihood exhibit slowing of primary brain rhythms,^31^ increased neuronal synchronization,^16^ as well as reduction of long-range temporal correlations and hyperexcitability.^17^

In this study, we investigated how motor and cognitive functions evolve along the alpha-synucleinopathy continuum from prodromal to overt stages, and if individual clinical progression measures can be extracted. In addition, we observed whether the clinical course of individual prodromal patients is associated with presynaptic dopaminergic damage and HD-EEG-related measures at baseline.

To do so, we trained a clinical progression model using a large longitudinal cohort of iRBD patients (*n* = 766). We then investigate whether the obtained individual model parameters are associated with the conventional clinical progression milestone, that is, phenoconversion. In a subgroup of 49 patients with iRBD, we observed how cortical electrophysiological dysfunctions and presynaptic dopaminergic impairment at the first observation are related to a worse clinical progression.

## Materials and methods

### Dataset description

All iRBD patients were diagnosed following the International Classification of Sleep Disorders criteria, third edition (ICSD-3).^32^ Participants receiving treatment for cognitive dysfunction (i.e. cholinesterase inhibitors or memantine) or parkinsonism were excluded. The dataset resulted in 815 iRBD patients divided into the learning set (*n* = 766) and the blind set (*n* = 49) (Table 1). The learning set included 766 iRBD patients (166 female, 67.9 ± 7.4 years at the first visit) who underwent at least one clinical follow-up after the first visit (i.e. baseline). Of these, 427 participants (90 female, 68.8 ± 7.1 years at baseline) were enrolled in the ‘The risk FActoRs PREdictive of phenoconversion in idiopathic REM sleep behavior disorder: the Italian Study’ (FarPReSto) dataset (sponsored by the Italian academy of sleep medicine, AIMS), as of April 2025^33,34^; 227 (59 female, 65.5 ± 7.9 years at baseline) were enrolled from the Centre for Advanced Research in Sleep Medicine, Montreal, Canada, as of March 2025; and 112 iRBD patients (17 female, 69.0 ± 5.9 years at first visit) were included from the Parkinson’s Progression Markers Initiative (PPMI) database (https://www.ppmi-info.org, https://www.ppmi-info.org/study-design/study-cohorts), as of December 2023. After 44.6 ± 36.2 months from baseline, 104 iRBD patients in the learning set were diagnosed as PD (parkinsonism-first), 71 were diagnosed as DLB (dementia-first) and 10 were diagnosed as MSA.

**Table 1:** Summary of main demographics and clinical information of iRBD patients included in the learning and blind sets, and healthy control subjects

	Baseline	V1	V2	V3	V4	V5	V6	V7	V8+
**Learning set**									
Subjects, M/F	600/166	600/166	299/90	194/50	134/32	84/20	57/15	38/11	22/8
Age, years	67.9 ± 7.4	69.5 ± 7.5	69.4 ± 7.0	70.3 ± 6.9	71.2 ± 6.5	71.8 ± 5.9	72.8 ± 6.3	73.0 ± 5.4	73.7 ± 5.7
MoCA	25.4 ± 3.1	25.1 ± 3.7	25.2 ± 3.6	25.3 ± 3.4	25.4 ± 3.6	26.1 ± 3.3	25.5 ± 2.9	25.7 ± 3.0	26.5 ± 2.7
MDS-UPDRS-III	5.4 ± 5.8	7.4 ± 7.7	7.7 ± 8.2	8.6 ± 8.5	9.8 ± 10.2	10.2 ± 10.7	8.4 ± 7.6	9.7 ± 8.7	9.7 ± 10.4
**Blind set**									
Subjects, M/F	42/7	42/7	33/6	30/5	19/5	13/03	9/2	N/A	N/A
Age, years	68.5 ± 7.1	70.2 ± 7.1	72.1 ± 6.3	73.6 ± 6.0	74.3 ± 5.7	75.2 ± 5.5	74.3 ± 5.0	N/A	N/A
MoCA	24.6 ± 3.4	25.3 ± 3.3	23.4 ± 4.5	21.7 ± 5.9	21.0 ± 7.3	20.2 ± 6.5	22.1 ± 6.0	N/A	N/A
MDS-UPDRS-III	0.7 ± 1.3	2.4 ± 4.1	4.8 ± 8.4	8.1 ± 12.2	10.4 ± 12.5	14.5 ± 13.5	12.8 ± 16.1	N/A	N/A
**Control set**									
Subjects, M/F	119/69	119/69	108/66	103/64	95/59	79/47	67/41	54/36	45/30
Age, years	62.3 ± 11.1	63.3 ± 11.2	64.2 ± 11.1	65.1 ± 11.0	66.1 ± 11.3	67.9 ± 11.5	68.7 ± 11.3	68.8 ± 11.4	69.5 ± 10.1
MoCA	27.3 ± 2.2	27.2 ± 2.4	27.4 ± 2.2	27.6 ± 2.2	27.7 ± 2.1	27.7 ± 2.2	27.9 ± 2.2	27.9 ± 2.2	27.8 ± 2.5
MDS-UPDRS-III	1.7 ± 2.9	1.7 ± 3.0	1.7 ± 4.0	2.1 ± 4.1	2.7 ± 4.2	2.7 ± 4.4	3.2 ± 5.1	3.1 ± 4.5	2.8 ± 4.3

Fluctuation within time points in the MDS-UPDRS-III mean estimates could be due to differences in the number of subjects and follow-up durations across the cohort (Supplementary Table 1). Note that rolling recruitment is used in all cohorts, so shorter duration of follow-up does not imply loss to follow-up. F = female; iRBD = idiopathic/isolated REM sleep behaviour disorder; N/A = not applicable; M = male; MDS-UPDRS-III = Movement Disorders Society–Unified Parkinson’s Disease Rating Scale Part III; MoCA = Montreal Cognitive Assessment.

The blind set included 49 iRBD patients (seven female, 68.5 ± 7.1 years at baseline), enrolled at the Sleep Lab, IRCCS Policlinico San Martino, Genoa, Italy. After 43.3 ± 28.0 months from baseline, 20 patients developed PD (*n* = 10) or DLB (*n* = 10). These patients underwent longitudinal clinical assessment, as well as baseline HD-EEG and DaT-SPECT.

As a control dataset, we included 188 healthy control subjects (69 female, 62.27 ± 11.14 years at baseline) who were enrolled from the PPMI database (Table 1).

For the following analysis, we defined non-converter iRBD (ncRBD) patients as those who were still free from parkinsonism/dementia at the last available clinical follow-up, and converter iRBD (cRBD) patients as those who developed PD, DLB or MSA.

Only for the PPMI cohort, we included the biological characterization (Supplementary Table 2) for further analysis, which will be useful for future clinical trials. At baseline, 80 iRBD patients were alpha-synuclein (alpha-syn) biomarker positive (age 69.6 ± 5.3 years at baseline, 13 female), 78 iRBD patients were hyposmic (age 69.7 ± 5.1 years at baseline, 12 female) and 11 iRBD patients had mild cognitive impairment (age 68.3 ± 5.8 years at baseline, five female).

All patients signed informed consent before participating in the study in compliance with the Helsinki Declaration of 1975.

Inclusion and exclusion criteria used by the FarPReSto consortium to collect the data are reported in Puligheddu *et al*.^33^ The eligibility criteria used by the PPMI can be found at https://www.ppmi-info.org/study-design/study-cohorts. The inclusion and exclusion criteria used by the Centre for Advanced Research in Sleep Medicine (Montreal, Canada) are reported in Postuma *et al*.^35^

### Clinical evaluation

All iRBD patients and healthy subjects underwent cognitive and motor assessment in at least two subsequent follow-ups. The clinical evaluation included the Unified Parkinson’s Disease Rating Scale Part III (UPDRS-III) or MDS-UPDRS-III to rate motor impairment, and the Mini-Mental State Examination (MMSE) or Montreal Cognitive Assessment (MoCA) as measures of global cognition.

We chose the MoCA and MDS-UPDRS-III scores in the following analysis to assess cognitive and motor impairment. Hence, we converted MMSE to MoCA^36^ and the UPDRS-III to MDS-UPDRS-III^37^ for all patients.

### Molecular imaging evaluation

Within 3 months following diagnosis, 49 iRBD patients (blind set) underwent brain DaT-SPECT according to international guidelines.^13^ Details on SPECT data acquisition and analysis are reported in the Supplementary material. After the acquisition, reconstructed images were processed by Basal Ganglia V2 software^38^ to compute specific non-displaceable binding ratios (SBRs). We subtracted the background uptake (occipital region) from putamen or caudate uptake as follows: (putamen/caudate uptake-background uptake)/background uptake. We computed the mean SBR values between the right and left hemispheres for the caudate and putamen for the following analyses.

### HD-EEG acquisition, preprocessing and analysis

Within 3 months following diagnosis, 49 iRBD patients (blind set) underwent HD-EEG during the morning to reduce drowsiness. The HD-EEG protocol consists of ∼20 min of wakeful resting-state recording using a Galileo system (EBNeuro) with 64 electrodes positioned according to the 10–10 international system. Each recording session includes eye-closed, eye-open and hyperventilation conditions. The sampling rate of recording signals was 512 Hz. During the EEG recording sessions, an EEG technician monitored the session to prevent patients from sleeping and maintain their vigilance. To remove the power line noise (50 Hz), we applied a Notch Filter (order 2). We rejected artefactual channels. To remove artefacts, we applied both independent component analysis (ICA) and visual inspection. We band-pass filtered the EEG time series with a broad-band Finite Impulse Response (FIR) filter (1–80 Hz, Kaiser window, order 1858) and interpolated bad channels using the spline interpolation method (kernel size: 4 cm). Finally, we applied the scalp current density (SCD) to all clean sensors with the spline method (lambda 0.00001, order 4, degree 14). For the following analysis, we used the eye-closed condition (11.48 ± 3.91 min).

After preprocessing, we analysed the broadband eye-closed SCD time series through a time-frequency decomposition using 30 log-spaced narrowband Morlet wavelets (m = 5) in the 2–70 Hz range.^39,40^ For each time-frequency series, we first quantified the phase synchronization with the weighted phase-lag index (wPLI)^41^ and the amplitude correlation using the orthogonalized correlation coefficient (oCC),^42^ respectively. Both wPLI and oCC ranged into [0, 1], where 0 indicates the absence of synchronization/correlation, and 1 means full synchronization/correlation. Second, we quantified the scale-free decay of temporal correlations in the amplitude modulation of neuronal oscillations^43^ with the detrended fluctuation analysis (DFA) scaling exponent (i.e. the slope of the fluctuation function). A scaling exponent in the range [0.5, 1] indicates power-law scaling behaviour and the presence of long-range temporal correlations (LRTCs), while an exponent below 0.5 is characteristic of an uncorrelated signal. Third, we evaluated the E/I balance by computing the functional excitation-inhibition ratio (fEI).^44^ An fEI = 1 means that the brain works in a perfect balance between excitation and inhibition, while an fEI greater or smaller than 1 indicates hyperexcitability or higher inhibition, respectively. Finally, we estimated the bistability index (BiS) to observe the discontinuous transition between asynchronous and fully synchronous activity.^23,26^ A BiS close to zero means that the single-exponential model is a more likely model for the observed time series, whereas, for a higher BiS, the most likely model for the observed time series is the bi-exponential model.

For the following analysis, we grouped the whole spectrum of each EEG measure in four frequency bands: delta (1–4 Hz), theta (4–8 Hz), alpha (8–13 Hz) and beta (13–20 Hz).

### Disease Course Mapping model

The DCM model is mapped to the individual data using three parameters: time-shift (τ), acceleration (α) and intermarker spacing (ω).^10^ The time-shift indicates if the temporal onset of the individual progression is earlier or later than the population progression. The acceleration factor shows how fast (α > 1) or slow (α < 1) the progression of subjects is compared with the population progression. The combination of time-shift and acceleration factors enables plotting longitudinal observations along a common trajectory by mapping chronological age to a latent ‘disease age’ timeline. Finally, the intermarker spacing specifies if one biomarker changes earlier (ω < 0) or later (ω > 0) than the other biomarkers for each subject. In this work, the intermarker spacing values indicate if the MDS-UPDRS-III changes earlier/later than the MoCA. To train the DCM model, we used LEarned Spatiotemporal patterns in Python (Leaspy v.1.5.0).^45^

### Experimental design

This study was divided into three phases (Fig. 1). In the first phase (Fig. 1A), we used the MDS-UPDRS-III and MoCA from the learning set (*n* = 766) to train an iRBD course map using the DCM model. Since the DCM model requires that all features are monotonically increasing, we inverted the values for MoCA. We normalized all model inputs (i.e. MoCA and MDS-UPDRS-III) between 0 and 1, where 0 indicated the minimum normal score value and 1 indicated the maximum pathological change. We split our dataset into a training set (80%) and a test set (20%) using a stratified 5-fold cross-validation: (i) to reduce the bias in modelling; and (ii) to have the same proportion of non-converter and converter iRBD patients in the training and test sets. For each fold, the procedure included: (i) calibration of the model on the training data and the estimation of the average progression trajectory for both biomarkers; (ii) the model personalization for a test subject with *n* observations by positioning the first *n* – 1 observations on the disease course map to obtain individual trajectory and model parameters (τ, α, ω); and (iii) the prediction of the future values and the evaluation of the prediction error by comparing the predicted and the original values. We averaged across folds the progression trajectory of the MDS-UPDRS III and MoCA. Moreover, we trained a DCM model using the MDS-UPDRS-III and MoCA from healthy subjects to observe whether progression of clinical scores in iRBD differed from changes expected with normal ageing.

**Figure 1:**
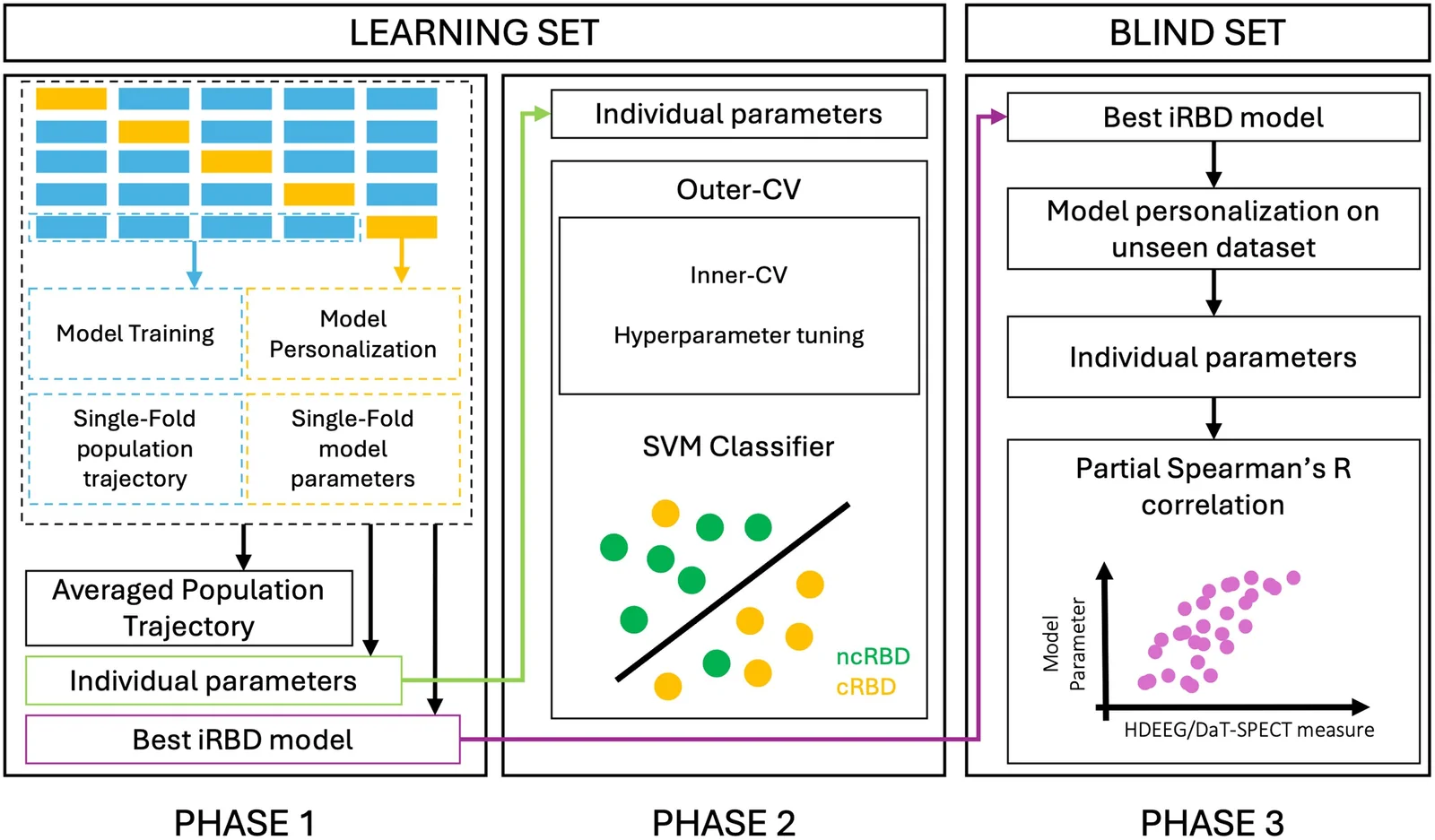
**Experimental design**. Phases 1 and 2 were carried out on the learning set (*n* = 766). Phase 3 was performed on the blind set (*n* = 49). CV = cross-validation; HDEEG = high-density-EEG; iRBD = idiopathic/isolated REM sleep behaviour disorder; SVM = support vector machine.

In the second phase (Fig. 1B), we carried out ncRBD/cRBD classification by using as input the single-subject model parameters (τ, α, ω). We performed a nested cross-validation approach by splitting the dataset (*n* = 766) into a learning and a testing set by a stratified 5-fold cross-validation (outer-CV), and then further splitting the learning set into training and validation sets by a stratified 5-fold cross-validation (inner-CV). The outer-CV allows testing the model with the best hyperparameter on an unseen dataset (testing set), while the inner-CV allows tuning the model hyperparameter. For each outer fold, we performed data standardization by computing the *Z*-score on the learning set and applying the same transformation to the test set. We applied the Synthetic Minority Over-sampling Technique (SMOTE) to the learning set by increasing the sample size in the minority class. Then, we trained a support vector machine (SVM) by searching for the best parameters (inner-CV): regularization parameter (among 1, 10, 100, 1000) and kernel type (among linear, poly, rbf and sigmoid). We selected the best hyperparameters that produced the lowest prediction error on the validation set [considering both f1-score and Matthew’s correlation coefficient (MCC): if the two metrics disagreed, we chose the best hyperparameters based on MCC alone]. Subsequently, we evaluated the best model performance in terms of accuracy, sensitivity, specificity, f1-score and MCC on the unseen testing set. This analysis is aimed at evaluating whether the extracted individual clinical progression parameters (τ, α, ω) are associated with the established clinical progression milestone, that is, phenoconversion.

In the third phase (Fig. 1C), we investigated whether the clinical progression markers extracted for the DCM model (τ, α, ω) are associated with baseline DaT-SPECT data, as an established nigro-striatal neurodegeneration biomarker, and with baseline HD-EEG metrics, as a potential cortical neurodegeneration biomarker. We used the clinical evaluation of the blind set (*n* = 49) to personalize the model predictions by obtaining the individual trajectory and model parameters (τ, α, ω) for each subject in the blind set. We computed the partial Spearman *R* correlation coefficient (PSCC) between model parameters and: (i) DaT-SPECT measures; and (ii) HD-EEG metrics, using age and sex as covariates. To correct the effect of multiple comparisons, we applied the Benjamini–Hochberg (BH) method (ɑ = 95%) across putamen and caudate or frequency bands (i.e. delta, theta, alpha and beta bands).^46^ This analysis is aimed at evaluating whether the extracted individual clinical progression parameters (τ, α, ω) are associated with the neurodegeneration biomarkers, to explore the biological correlates of these metrics.

### Statistical analysis

We evaluated the accuracy of the model prediction by quantifying the mean and the standard deviation of the mean absolute error between predicted data and real data across folds for MDS-UPDRS III and MoCA. Age and sex were used as nuisance variables in all statistical analyses involving correlations between model parameters and biomarkers (both DaT-SPECT and EEG).

We measured the probability that phenoconversion occurs over time by using the cumulative incidence curve.

We investigated whether there were differences in acceleration factor, time-shift and intermarker spacing between non-converters and healthy controls, non-converter and converter iRBD patients, and between PD-converter and DLB-converter iRBD patients. Owing to the small number of MSA-converters, this group was excluded from the PD- versus DLB-converter analysis. Pairwise differences in model parameters were assessed using a two-tailed Wilcoxon rank-sum test (α = 0.05), with BH correction for multiple comparisons. Only for the PPMI cohort, we investigated whether biological stratification at baseline (i.e. alpha-syn biomarker, olfactory function and cognitive status) impacted disease progression trajectory in the iRBD population. All pairwise differences were assessed using a Wilcoxon rank-sum test (α = 0.05). All *P*-values were corrected with the BH method across model parameters.

### Sample size calculation

Sample size was estimated by treating the subject-specific time-shift (τ) and acceleration (α) parameters, derived from the disease progression model, as continuous outcomes. Using the variability of each parameter observed in the study population, we calculated the number of participants required in each group to detect a prespecified difference in mean time-shift or acceleration between groups, with a given statistical power and significance level. This approach allows sample size requirements to be derived directly from the estimated dispersion of the DCM model parameters, and the minimum differences considered clinically meaningful.

## Results

### Motor testing worsens faster than cognitive testing in iRBD patients

Using the DCM model, we trained a disease course map of MoCA and MDS-UPDRS-III scores to investigate the changes over time in cognitive and motor functions in the iRBD population (Fig. 2). The iRBD model forecasts the clinical scores with an averaged mean absolute error across folds of 0.041 for MDS-UPDRS-III (min: 0.032 and max: 0.047) and 0.071 for MoCA (min: 0.066 and max: 0.075). In addition, the mean slope of the MDS-UPDRS-III sigmoid was 0.005, whereas it was 0.0035 for the MoCA sigmoid. The MDS-UPDRS-III sigmoid was 35% faster than the MoCA sigmoid, which means that the motor score progressed more quickly than the cognitive score. This observation may partially reflect the lower sensitivity of the MoCA to subtle longitudinal changes, as well as a potential administration bias, rather than a true slower cognitive decline.^6,35,47^ Notably, a faster motor worsening is not necessarily related to the earlier emergence of overt parkinsonism that requires the presence of bradykinesia with resting tremor and/or rigidity for diagnostic purposes. Finally, we compared the probability of occurrence of phenoconversion, which are standard clinical milestones, with the clinical disease progression trajectory (Fig. 2C). As expected, our results showed that the DCM trajectory indicated a decline in motor and cognitive scores 10–15 years before the phenoconversion event (>20%). This finding confirms the notion that the individual clinical progression scores worsen earlier than the occurrence of a definite phenoconversion diagnosis, best reflecting the disease progression.

**Figure 2:**
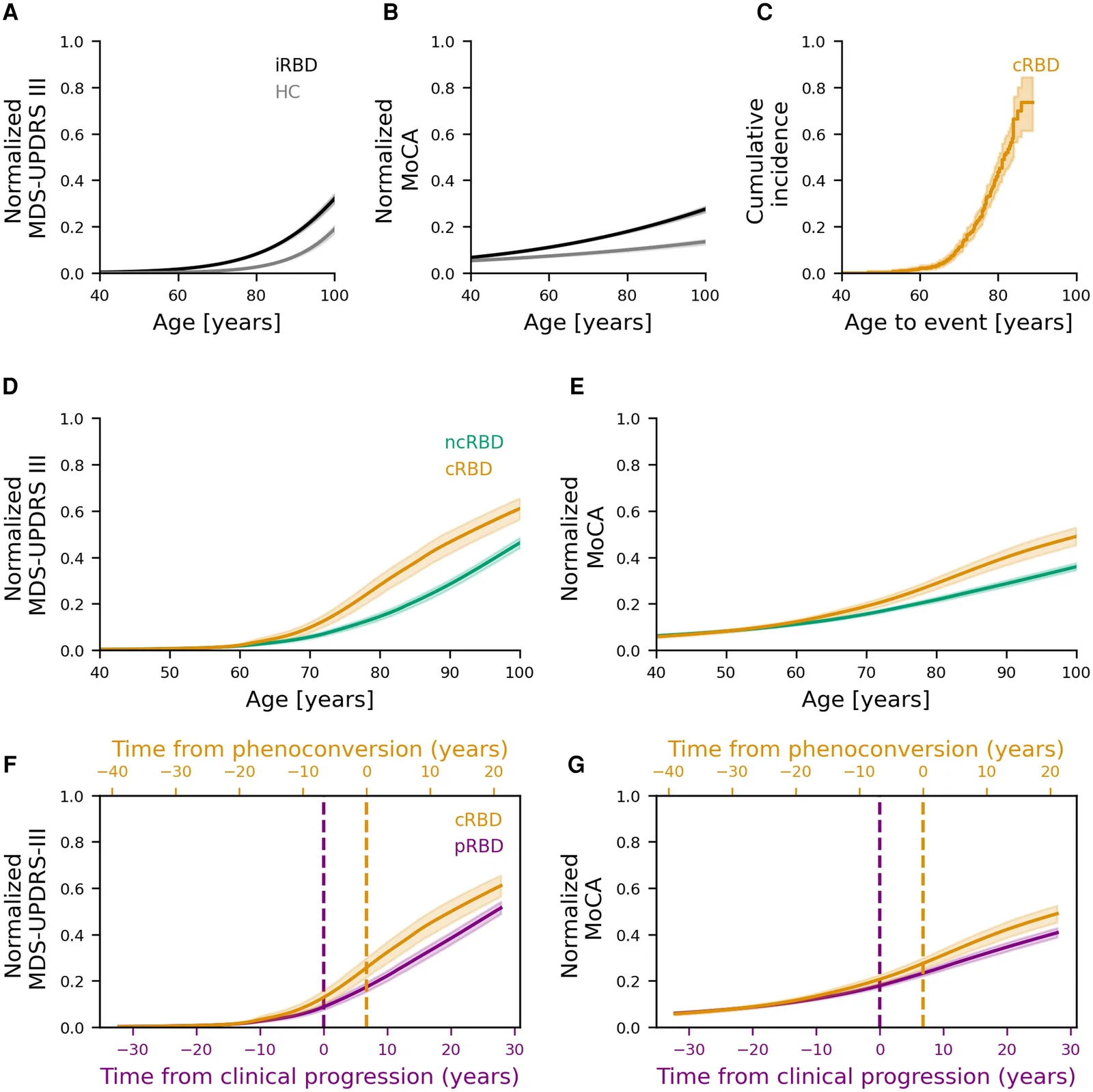
**MDS-UPDRS-III and MoCA progression across time of iRBD patients and healthy controls**. Average over 5-fold of (**A**) MDS-UPDRS-III and (**B**) MoCA progression trajectories for iRBD population (black) and healthy controls (grey). (**C**) Cumulative incidence curve of phenoconversion probability (orange). The shaded area indicates the pointwise confidence interval of the Kaplan–Meier estimator. Personalized (**D**) MDS-UPDRS-III and (**E**) MoCA progression trajectories for non-converter (green) and converter (orange) iRBD patients. Personalized (**F**) MDS-UPDRS-III and (**G**) MoCA progression trajectories of converter iRBD patients aligned to phenoconversion event (orange) and aligned to clinical motor/cognitive progression (purple). (**A**–**G**) The shaded areas represent 5% confidence intervals around the population mean (bootstrap, *n* = 1000). cRBD = converter iRBD; HC = healthy controls; iRBD = idiopathic/isolated REM sleep behaviour disorder; MoCA = Montreal Cognitive Assessment; MDS-UPDRS-III = Movement Disorder Society–Unified Parkinson’s Disease Rating Scale, motor section; ncRBD = non-converter iRBD; pRBD = progressed iRBD.

Despite the aforementioned limitations, we performed an additional analysis by aligning personalized iRBD trajectories with the phenoconversion event and the motor/cognitive progression event. The motor progression event was defined as an increase of at least 4 points on the MDS-UPDRS-III, while a cognitive progression event was defined as an increase of at least 3 points on the MoCA.^6^ According to these rules, our dataset included 185 converter iRBD (age 68.2 ± 6.8 years at baseline, 46 female) and 581 non-converter iRBD (age 67.8 ± 7.6 years at baseline, 120 female) when using the standard phenoconversion criteria. Additionally, 522 patients (age 68.0 ± 7.1 years at baseline, 114 female) met the clinical progression event (either motor or cognitive), while 244 patients (age 67.6 ± 8.0 years at baseline, 52 female) did not. Then, we aligned personalized iRBD trajectories to both the phenoconversion and the clinical progression events. This analysis suggests that the onset of motor/cognitive progression occurs 6.8 years before the phenoconversion event on the latent disease timeline (Fig. 2F and G).

### RBD evolution is different from healthy ageing

Using DCM model parameters, we tested whether the progression of clinical scores in the non-converter iRBD patients differed from that due to normal healthy ageing. First, we trained a separate model using the healthy subjects available within PPMI (*n* = 188, Fig. 2A and B). Then, we tested whether their model parameters (τ, α, ω) were significantly different from those estimated on ncRBD patients (Supplementary Fig. 1). Our results indicated that ncRBD patients showed, on average, a significant 4.68-year earlier decline (*P* = 5.39 × 10^−21^, Wilcoxon rank-sum test, BH corrected) and 10% faster initial progression (*P* = 0.01) with a strong to small effect size (rank biserial correlation – RBC = 0.46 and −0.13) (Supplementary Fig. 1). Altogether, these results suggest that the trained model captures the progression of the clinical scores and that the model parameters differ from healthy ageing.

### Differences in clinical profiles by phenotype

We aimed to observe differences in symptom profiles between non-converter (*n* = 581) and converter (*n* = 185) iRBD patients in the learning set (Figs 2C, D and 3A). At the baseline visit, non-converters significantly differ from converters (Wilcoxon rank-sum test, BH corrected) for both MDS-UPDRS-III (*P* = 0.0007) and MoCA (*P* = 0.002), but the effect size indicates a negligible small effect (Δ_MDS-UPDRS-III_ = 1.04, Δ_MoCA_ = −0.08) (Table 2 and Supplementary Fig. 2). Acknowledging the large variance in follow-up time points in the study sample, we compared clinical data at baseline of patients with one follow-up only (*n* = 377) versus the remaining dataset (*n* = 389). Our data showed no significant differences (*P*_MoCA_ = 0.11, *P*_MDS-UPDRS-III_ = 0.06, Wilcoxon rank-sum test, BH corrected). Subsequently, we observed the difference between the two groups in the model parameters (i.e. time-shift, acceleration factor and intermarker spacing). As expected, converter iRBD patients exhibited ∼30% faster (Δα=−0.27, *P_α_* = 0.00002, Wilcoxon rank-sum test, BH corrected) and 2.14-year earlier onset of clinical worsening than non-converter iRBD patients (*P*_τ_ = 0.000016, Wilcoxon rank-sum test, BH corrected) with a small to medium effect size (Table 2). Motor score worsened earlier than the cognitive one in converter iRBD patients (*P*_ω_ = 0.027, Wilcoxon signed-rank test, BH corrected) with a weak effect size (Table 2). Notably, we found that the model parameters could discriminate between non-converter and converter iRBD patients with an accuracy of 73% (f1-score: 73% and MCC: 0.27) (Table 3).

**Figure 3:**
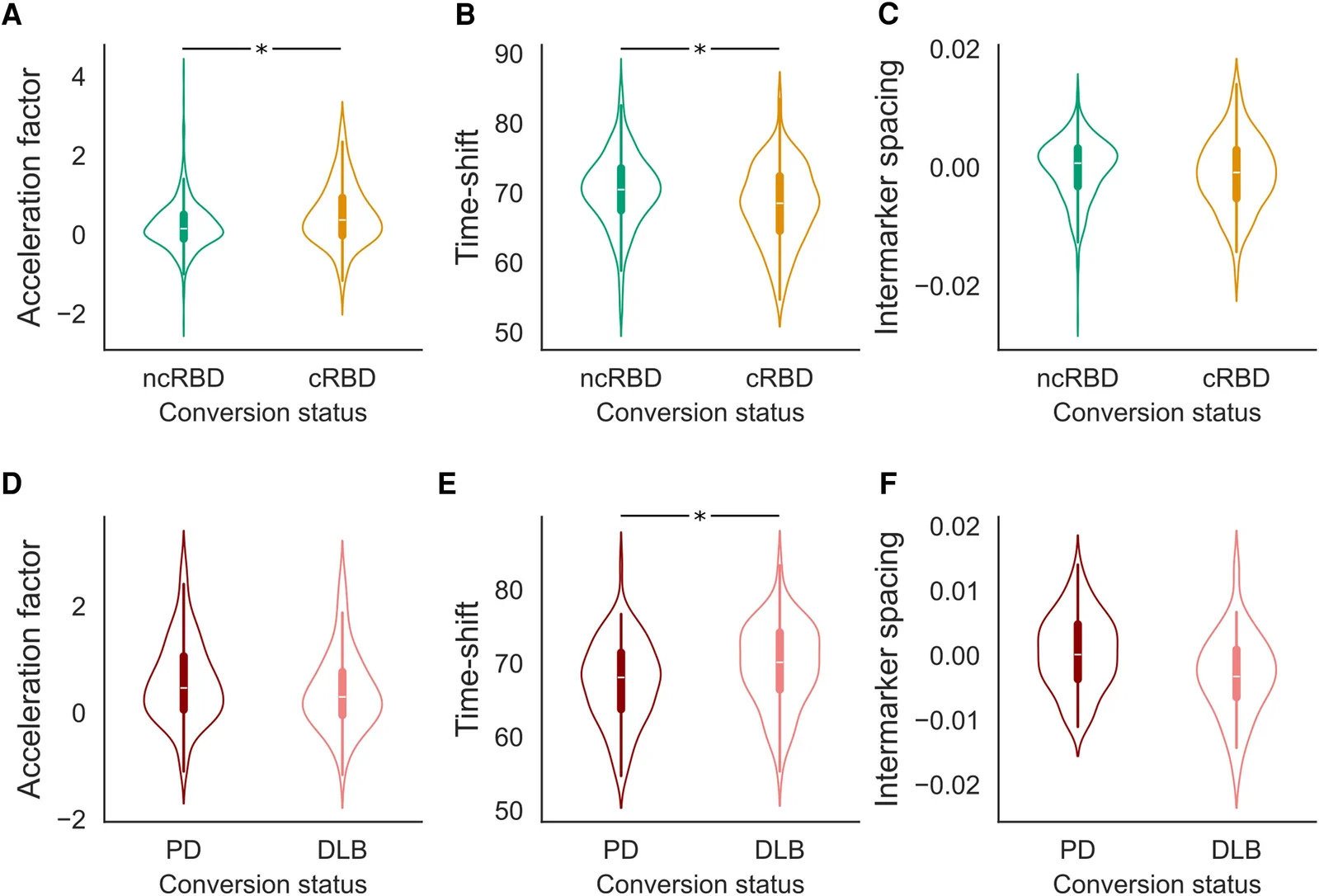
**Converter iRBD patients exhibit a worse clinical scenario**. Violin plot of acceleration factor (**A**), time-shift (**B**) and intermarker spacing (**C**) for non-converter (green) and converter (orange) iRBD patients. Violin plot of acceleration factor (**D**), time-shift (**E**) and intermarker spacing (**F**) for PD-converter (dark red) and DLB-converter (light red) iRBD patients. The asterisk indicates a significant difference between the two populations (Wilcoxon rank-sum test, BH corrected). BH = Benjamini–Hochberg; DLB = dementia with Lewy bodies; iRBD = idiopathic/isolated REM sleep behaviour disorder; PD = Parkinson's disease

**Table 2:** Summary statistics

	Variable	*P*	RBC	95% CI	*P*(X > Y)
cRBD versus ncRBD	MoCA	0.002	0.15	0.05; 0.25	58%
	MDS-UPDRS-III	0.0007	−0.18	−0.27; −0.08	41%
cRBD versus ncRBD	*t*	0.000016	0.22	0.12; 0.32	61%
	*a*	0.000022	−0.21	−0.31; −0.11	39%
	*w*_UPDRS, ncRBD_	0.027	0.11	0.01; 0.21	55%
RBD versus HC	*t*	5.39 × 10^−21^	0.46	0.39; 0.58	73%
	*a*	0.01	−0.13	−0.23; −0.03	43%
	*w*	0.22	−0.06	−0.14; 0.02	47%
PD versus DLB	*t*	0.01	0.71	0.07; 0.41	86%
	*a*	0.09	0.56	−0.32; 0.02	78%
	*w*	0.001	0.50	−0.48; −0.15	75%

CI = confidence interval; cRBD = converter iRBD; DLB = dementia with Lewy bodies; HC = healthy controls; MDS-UPDRS-III = Movement Disorder Society–Unified Parkinson’s Disease Rating Scale motor section; MoCA = Montreal Cognitive Assessment; ncRBD = non-converter iRBD; *P*(X > Y) = probability of measuring a larger effect X than Y; PD = Parkinson’s disease; RBC = rank biserial correlation.

**Table 3:** Summary of non-converters/converters classifier performance

	Performance, mean ± SD	Fold 1	Fold 2	Fold 3	Fold 4	Fold 5
Accuracy	0.73 ± 0.02	0.75	0.75	0.73	0.68	0.72
Specificity	0.54 ± 0.04	0.53	0.63	0.51	0.51	0.54
Sensitivity	0.73 ± 0.02	0.75	0.75	0.73	0.68	0.72
f1-score	0.73 ± 0.02	0.75	0.76	0.72	0.69	0.72
MCC	0.27 ± 0.06	0.30	0.36	0.24	0.19	0.25

MCC = Matthew’s correlation coefficient; SD = standard deviation.

We then investigated if there was a difference between iRBD patients who first developed PD (*n* = 104) and those who first developed DLB (*n* = 71) (Fig. 3D–F). PD-converter patients exhibited clinical worsening, on average, 2.3 years earlier than DLB-converter patients (*P*_τ_ = 0.01). Interestingly, the motor score worsened earlier than the cognitive score in DLB-converter patients (*P*_ω_ = 0.001, Wilcoxon signed-rank test, BH corrected) with a strong effect size (Table 2).

### Differences in clinical profiles by biological characterization

We aimed to explore whether biological stratification (i.e. alpha-syn biomarker, olfactory function and cognitive status) affected disease progression (Supplementary Table 3 and Supplementary Fig. 3). We found a small, statistically significant increase in acceleration between iRBD patients with the alpha-syn biomarker compared with those without it (RBC_α_ = −0.29, *P_α_* = 0.016, Wilcoxon rank-sum test, BH corrected). Likewise, acceleration tended to be higher in hyposmic than normosmic iRBD patients (RBC_α_ = −0.24, *P*_α_ = 0.049) and in iRBD patients with mild cognitive impairment than those with normal cognition (RBC_α_ = −0.23, *P*_α_ = 0.218); however, these differences were not significant. The results of this subanalysis should be considered exploratory, considering the limited sample size.

### Nigro-striatal dopaminergic impairment is linked to faster disease progression

We explored if model parameters (τ, α and ω) correlated with baseline nigro-striatal dopaminergic function in the 49 iRBD patients included in the blind set. The time-shift was directly correlated with the SBRs at putamen (ρ = 0.53, *P* = 0.0002, BH corrected) and caudate levels (ρ = 0.39, *P* = 0.0061, BH corrected) (Fig. 4). The acceleration factor tended to be inversely correlated with the SBRs in the putamen (ρ = −0.26, *P* = 0.07, BH corrected) and caudate (ρ = −0.31, *P* = 0.07, BH corrected) levels (Fig. 4). There was no significant correlation between SBRs and intermarker spacing (Fig. 4). These results suggest that low DaT levels at baseline are associated with earlier and faster clinical worsening.

**Figure 4:**
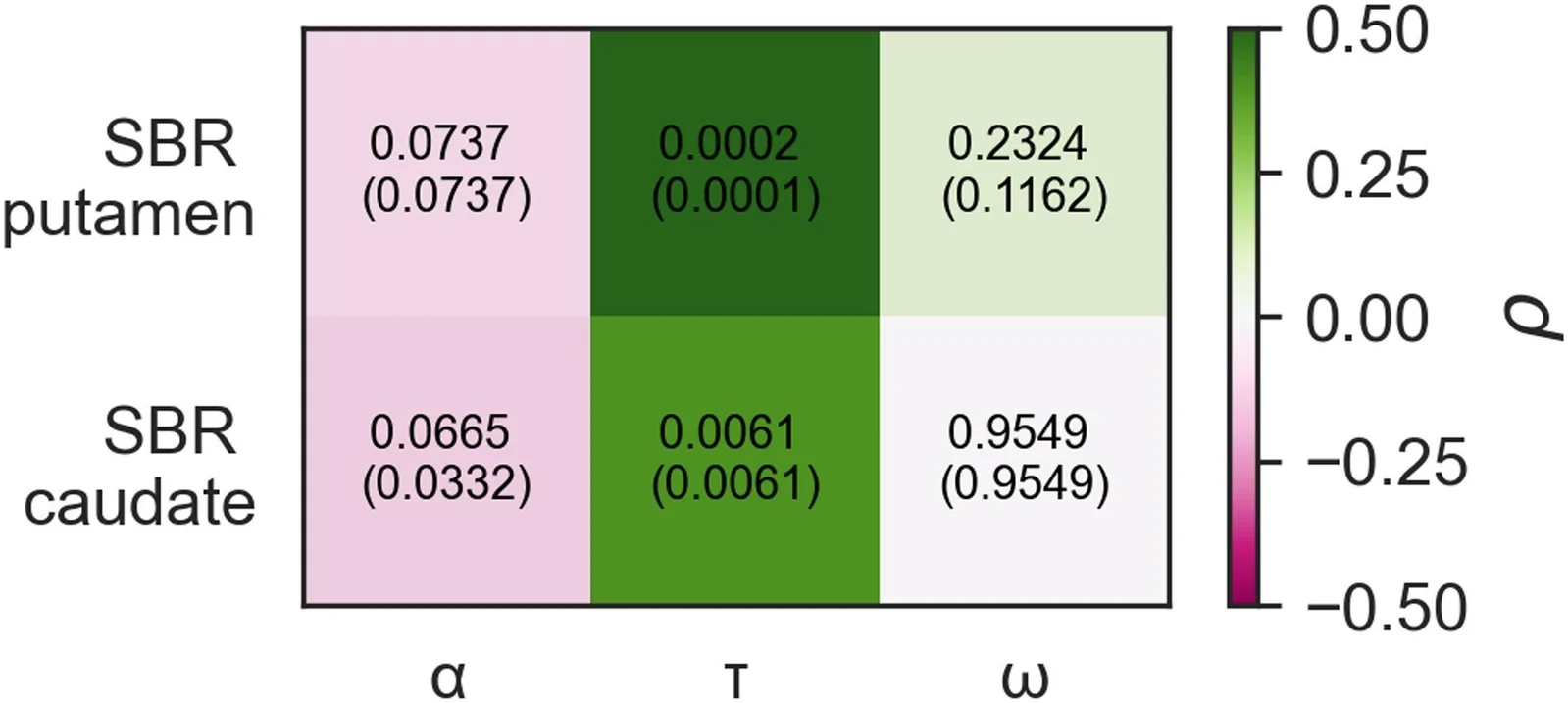
**Nigro-striatal dopaminergic impairment is related to faster disease progression**. Spearman’s *R* correlation between model parameters and DaT levels in the putamen and caudate. The colour bar reports the correlation coefficient values. The pixel transparency indicates if the *P*-value is significant after Benjamini–Hochberg (BH) correction (no transparency) or not (70% transparency). The numbers indicate the *P*-values after BH correction, while the numbers in parentheses indicate the *P*-values before BH correction. DaT = dopamine transporter; SBR = specific non-displaceable binding ratio.

### Altered large-scale network interactions are related to faster disease progression

We aimed to investigate if baseline alterations of critical dynamics, E/I balance and large-scale network interaction were related to the clinical metrics of disease progression in patients with iRBD. The wPLI in the theta band was negatively and significantly correlated with the time-shift (ρ = −0.37, *P* = 0.045, BH corrected, Fig. 5B) and the intermarker spacing (ρ = −0.46, *P* = 0.004, BH corrected, Fig. 5C). Acceleration factors positively correlated, but not after BH correction, with wPLI in the theta band (ρ = 0.29, *P* = 0.192, BH corrected), oCC in the delta band (ρ = 0.31, *P* = 0.133, BH corrected), fEI (ρ = 0.31, *P* = 0.145, BH corrected) and DFA scaling exponent in the delta band (ρ = 0.29, *P* = 0.196, BH corrected) (Fig. 5A).

**Figure 5:**
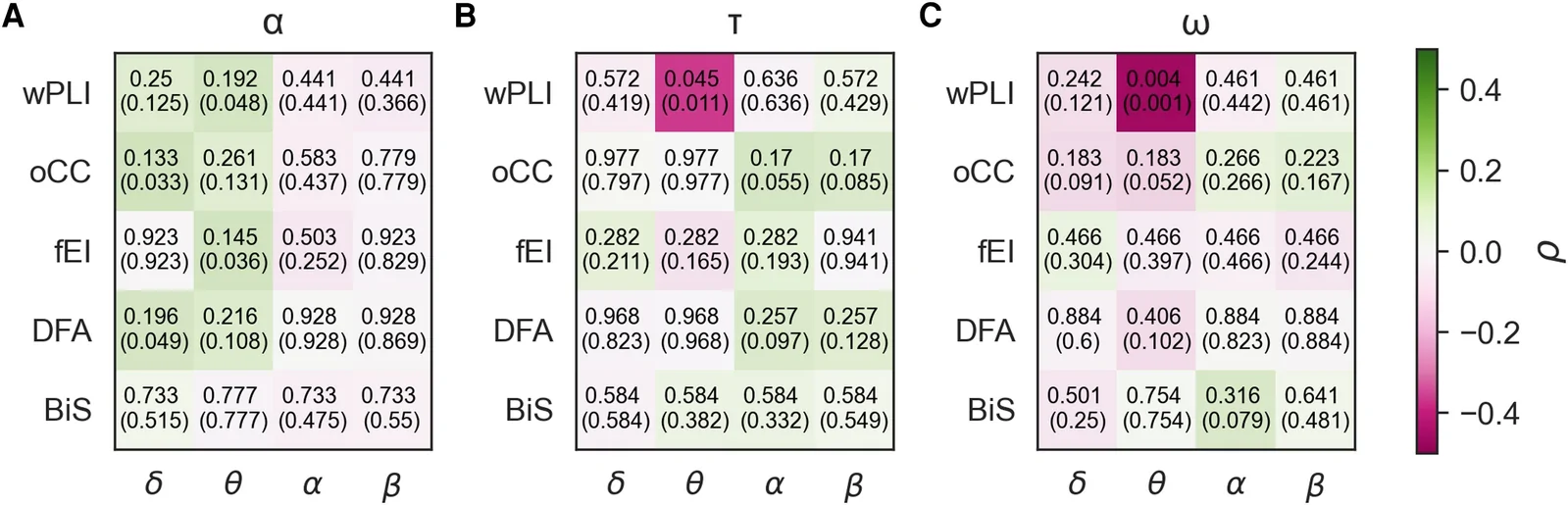
**Higher synchronization is related to worse disease progression.** Spearman’s rank correlation coefficient between EEG measures (grouped in canonical frequency bands) and (**A**) acceleration factor, (**B**) time-shift and (**C**) intermarker spacing. The colour bar reports the correlation coefficient values. The pixel transparency indicates if the *P*-value is significant after Benjamini–Hochberg correction (no transparency) or not (70% transparency). The numbers indicate the *P*-values after BH correction, while the numbers in parentheses indicate the *P*-values before Benjamini–Hochberg correction. BiS = bistability index; DFA = detrended fluctuation analysis; fEI = functional excitation-inhibition ratio; oCC = orthogonalized correlation coefficient; wPLI = weighted phase-lag index.

This finding suggests that patients with iRBD with higher synchronization at baseline have an earlier neurodegeneration process and, especially, earlier motor impairment.

### Sample size calculation

On average, converter iRBD patients showed an earlier progression by 2.14 years and a faster progression rate by 0.2 compared with non-converter iRBD patients. In a 1:1 treatment–control clinical trial designed to assess a drug’s ability to slow symptom progression, we would therefore expect an effect size comparable to, or potentially larger than, that observed in the overall iRBD population. Assuming equal variance between groups, a time-shift effect greater than 2 years, and a deceleration of 0.3 between converter and non-converter iRBD patients, with a significance level of α = 0.05 and 80% power, the required sample sizes are 101/101 patients per group for the time-shift analysis and 75/75 patients per group for the acceleration factor analysis (Supplementary Fig. 4).

## Discussion

In this longitudinal study involving a large cohort of patients with iRBD, we trained an advanced disease progression model, extracting individual metrics able to monitor clinical disease progression along the prodromal to overt alpha-synucleinopathy continuum. In detail, we identified three model parameters that are related to individual clinical characteristics representing early/late (time-shift, τ) and fast/slow (acceleration factor, α) progression, along with the individual trajectory (intermarker spacing, ω), which is earlier motor or cognitive worsening. These parameters demonstrate clinical meaningfulness as continuous indicators of disease progression, at the single-subject level, along the prodromal to overt alpha-synucleinopathy continuum.

To date, the most common clinical milestone of progression in the prodromal to overt alpha-synucleinopathy continuum is phenoconversion, that is, the emergence of overt parkinsonism or dementia. However, this binary approach is an oversimplification; the transition from prodromal to overt stages is progressive. Moreover, the diagnosis of overt syndromes is particularly user-dependent, with significant diagnostic instability, despite the presence of international guidelines.^48^ This is of paramount importance when designing disease-modifying clinical trials. Using phenoconversion as a primary end point may be unreliable, and reliable continuous progression metrics are needed.^49^

In the present study, we demonstrated that the parameters obtained from the disease progression model efficiently monitor motor and cognitive worsening along the prodromal to overt alpha-synucleinopathy continuum. Their clinical relevance is further supported by the observed correlations with both DaT-SPECT and HD-EEG, confirming that these model-derived metrics reflect underlying pathophysiological mechanisms. Converter-RBD patients have an earlier and faster clinical worsening, as characterized by the individual model parameters (time-shift, τ, and acceleration factor, α). Notably, the model parameters correctly identified converter-RBD patients with an accuracy of 73%, highlighting their potential utility as end points in disease-modifying clinical trials. However, these metrics need to be validated in an independent, external cohort, and compared with other clinical metrics, to fully elucidate their clinical meaningfulness.

Our prediction performance is consistent with previous results.^14,50-53^ Nonetheless, after correction for multiple comparisons, only associations involving theta-band phase synchronization remained marginally significant. These imperfect associations between the model parameters and phenoconversion suggest that the two approaches assess different, partially overlapping clinical aspects. Therefore, these results should be considered exploratory and hypothesis generating rather than conclusive. Moreover, it should be acknowledged that part of the observed cognitive decline may reflect normal ageing effects. We hypothesize that the individual model metrics may best reflect the complexity of progression. That is, many patients with iRBD likely have mixed motor and cognitive features evolving concurrently or variably, and rigid overt syndrome diagnoses (i.e. PD, DLB and MSA) may not capture meaningful clinical worsening. For example, current PD diagnostic criteria require the presence of bradykinesia associated with resting tremor and/or rigidity.^3^ Thus, iRBD patients not fulfilling PD criteria may actually have higher MDS-UPDRS-III scores than some patients with a PD diagnosis (who, for example, may have mild bradykinesia and unilateral rigidity).

This finding has implications for designing disease-modifying trials. That is, using a meaningful clinical worsening of either motor or cognitive performance likely best reflects the overall clinical progression of patients across the prodromal to overt alpha-synucleinopathy continuum, compared with a binary and potentially rigid end point such as phenoconversion. This is in line with a recent large multicentric study showing that the combination of motor and cognitive worsening accurately monitors disease progression in patients with iRBD.^6^ According to previous literature,^54^ our results further suggest that clinical progression (i.e. motor and cognitive decline) occurs several years before the phenoconversion diagnosis. Thus, filling the research and clinical need for reliable, continuous progression metrics, in the present study, we provide individual metrics (time-shift, τ, acceleration factor, α, and intermarker spacing, ω) that can monitor either cognitive or motor worsening over time.

Another known limitation of using phenoconversion as the key outcome in the alpha-synucleinopathy continuum is the assumption that the time to phenoconversion from the prodromal stage (i.e. iRBD) to PD and DLB is the same. Our data showed that PD-converters had an earlier clinical worsening (i.e. lower time-shift, τ) compared with DLB-converters when the combination of motor and cognitive performances is assessed. Previous literature data suggest that the different timing of the onset of dementia and parkinsonism may distinguish DLB from PD.^2,6,55-60^ Notably, the average time to phenoconversion in PD-converters and DLB-converters is usually not statistically different at the group level, albeit with high variance.^2,6,14,15^ Thus, monitoring the progressive clinical worsening with continuous metrics at the individual level instead of focusing on the time to overt syndrome diagnosis should overcome the risk of not achieving a rigid outcome, such as the phenoconversion, during the time frame of a clinical trial.

Aiming to achieve a comprehensive view of iRBD pathophysiology, it is crucial to include both cortical and subcortical changes. Here, we reported a correlation between nigro-striatal dopaminergic impairment at first observation and earlier as well as faster disease progression in our model. This result is in line with previous studies suggesting that nigro-striatal dopaminergic impairment is greater in iRBD patients with a high risk of short-term phenoconversion and may be an excellent biomarker to predict high-risk short-term phenoconversion into alpha-synucleinopathies in iRBD patients.^14,15,51,61^ Interestingly, we observed a strong correlation between neuronal synchronization at the first observation and a worse clinical scenario. This result supports the findings of recent studies showing that abnormalities in cortical neurophysiological dynamics might reflect an early neurodegeneration in the prodromal stage of alpha-synucleinopathies.^16-19^ Specifically, we reported that an earlier disease progression is related to higher phase synchronization in the theta (4–8 Hz) band. Previous literature findings reported that higher phase synchronization may reflect worsening of the biological disease severity in the iRBD population, characterizing those patients who phenoconverted into an alpha-synucleinopathy with disease progression.^17^ Here, we suggest that iRBD patients who present high phase synchrony at the first observation might have a high risk of developing alpha-synucleinopathy in a short time. In addition, a faster disease progression is partially positively related to higher phase synchronization and hyperexcitability in the theta (4–8 Hz) band, and with higher amplitude correlation and low values of LRTCs in the delta band (1–4 Hz). Thus, monitoring the global synchronization levels as early as the first observation, alongside longitudinal clinical evaluation, may be promising to provide new clues in the neurodegeneration process of iRBD populations. Overall, these results suggest that the extracted individual clinical progression markers are associated with both an established biomarker of substantia nigra neurodegeneration, as well as with an innovative biomarker reflecting brain cortex neurodegeneration.

We acknowledge that this work has some limitations. First, we only used the MoCA/MMSE and the MDS-UPDRS-III to assess longitudinal clinical change over time. These two tools are commonly used to measure global cognitive and motor functioning. However, it would be interesting to evaluate progression trajectories with a broader set of cognitive and motor biomarkers, as well as with other signs and symptoms commonly observed in alpha-synucleinopathies, such as biomarkers of autonomic function and smell tests. Furthermore, the MDS-UPDRS-III may be more sensitive to subtle changes than the MoCA or MMSE, and the cognitive tests are also limited by training effects. For this, the differences observed in the progression of the clinical scores might be due to the lower sensitivity of the MoCA. Second, despite the study including a large sample with longitudinal clinical assessment, longitudinal instrumental data (i.e. through DaT-SPECT or HD-EEG) were not available. Future studies should investigate whether the progression of clinical metrics is paralleled by the worsening of neurodegeneration biomarkers. Third, we provided individual parameters to monitor disease progression, but their use is currently hindered by the need for high-level computational expertise. The next step of the current project is to provide an open-source, user-friendly tool that would allow the application of these parameters in external cohorts or even individual subjects, without advanced computational knowledge. Fourth, the sensor-level EEG signals could be affected by volume conduction.^41,62^ Here, we used EEG metrics insensitive to estimate phase synchronization and amplitude correlation to avoid the volume conduction issue. Lastly, our cohort of iRBD patients mostly lacked biomarker confirmation for the presence of alpha-syn. Indeed, up to 30% of iRBD patients may be alpha-syn-negative.^52^ Our findings will need to be replicated in an independent cohort with confirmed alpha-syn biomarkers, to determine whether alpha-syn-negative participants have different progression rates. Moreover, we focused on iRBD patients as the prodromal alpha-synucleinopathy phenotype, but the present model should also be validated in other prodromal phenotypes, such as people with hyposmia or neurogenic orthostatic hypotension, and further validated, including external datasets.

In conclusion, this study provides new insights into the alpha-synucleinopathy continuum from the prodromal to the overt stage. We used both clinical and instrumental measures as neurodegeneration biomarkers to investigate the disease progression of a large iRBD population. We identified three individual clinical biomarkers (time-shift, τ, acceleration factor, α, and intermarker spacing, ω) that efficiently monitor disease progression in the prodromal to overt alpha-synucleinopathy continuum. These biomarkers are significantly associated with the established clinical progression milestone, that is, phenoconversion, but with the added value of being continuous variables, making them more suitable to more precisely monitor meaningful clinical worsening over time. We suggest that these metrics may be used as efficacy end points in disease-modifying clinical trials. Moreover, these individual markers are significantly associated both with presynaptic dopaminergic imaging, as an established substantia nigra neurodegeneration biomarker, and with neurophysiological activity, as an innovative brain cortex neurodegeneration biomarker, further highlighting the biological validity of the model.

## Supplementary Material

awag193_Supplementary_Data

## Data Availability

The data supporting the findings of this study can be obtained upon reasonable request. The iRBD progression model and the example code can be found at this link: https://github.com/MonicaRoascio/iRBDprogression.
