# Supplementary material for: Clinical markers of disease progression in the prodromal to overt alpha-synucleinopathy continuum
Source: Brain. 2026 May 29;149(10):3524–37. doi: 10.1093/brain/awag193 (PMC13634634; doi:10.1093/brain/awag193)
Supplement: Supplementary file 1 [file awag193_supplementary_data.pdf]

Supplementary materials

^{123}I-FP-CIT-SPECT image acquisition and reconstruction

We used a dual-head Millennium VG camera (GE Healthcare) equipped with low energy, high resolution, parallel-beam collimators to perform SPECT images. Scans were acquired 180-240 minutes after intravenous administration of 185 MBq of ^{123}I-FP-CIT (DaTSCAN, GE Healthcare, Little Chalfont, Buckinghamshire, UK), and lasted 40 minutes. We applied a "step-and-shoot" protocol with a radius rotation lower than 15 cm, generating 120 projections evenly spaced over 360°. Total counts were comprised of between 2 and 3 million. We used an electronic zoom (zoom factor = 1.8) during the data-collecting phase to obtain an acquisition matrix's pixel size of 2.4 mm. Subsequently, a digital zoom was applied during the reconstruction phase. The resulting images were sampled by cubic voxels (2.33 mm). We processed projections by applying an OSEM algorithm (8 interactions, 10 subsets) that included a proback pair accounting for collimator blur and photon attenuation, and then post-filtering (3-D Gaussian filter with full-width at half maximum 58 mm). Photon attenuation was modeled with the approximation of a linear coefficient uniform inside the skull and equal to 0.11 cm^{-1}, and a 2D+ 1 approximation was applied in the simulation of the space simulation blur. Compensation for scatter was not performed.

Basal Ganglia software functioning

Basal Ganglia software is an automatic algorithm based on a high-definition, 3-dimensional striatal template derived from the Talairach atlas. In detail, an automated algorithm performs fine adjustments in positioning blurred templates to match radioactive counts, meanwhile, it locates occipital regions of interest (ROI) to perform background evaluation. Also, a partial volume effect (PVE) correction is performed during the uptake computation of the putamen/caudate and background.

Supplementary tables

Tab. S1: Mean MDS-UPDRS-III score, MoCA score at baseline visit, and follow-up duration separately for the different cohorts

	FARPRESTO	MONTREAL	PPMI
**MDS UPDRS III**	4.89 ± 6.6	5.89 ± 4.4	4.57 ± 3.5
**MoCA**	24.82 ± 3.2	25.86 ± 2.8	26.53 ± 3.2
**Follow-up duration**	2.8 ± 2.5	4.8 ± 3.6	2.78 ± 2.6

***Table S2**:* PPMI cohort demographic information by biological characterization.

	**α-syn biomarker**		**Olfactory function**		**Cognitive status**	
	Negative	Positive	Normosmic	Hyposmic	Normal	MCI
**Subjects (M/F)**	28/4	67/13	29/5	66/12	89/12	6/5
**Age at baseline (years)**	67.5±7.0	69.6±5.3	67.5±7.3	69.7±5.1	69.1±6.0	68.3±5.8

***Table S3**:* Summary statistics.

	**α-syn biomarker**			**Olfactory function**			**Cognitive status**		
	RBC	*p*	*p* corrected	RBC	*p*	*p* corrected	RBC	*p*	*p* corrected
**Acceleration factor**	-0.29	0.016	0.048	-0.24	0.049	0.146	-0.23	0.218	0.327
**Time-shift**	-0.06	0.613	0.613	0.02	0.857	0.947	0.16	0.390	0.390
**Intermarker spacing**	-0.06	0.595	0.613	0.01	0.947	0.947	0.41	0.026	0.079

Supplementary figures

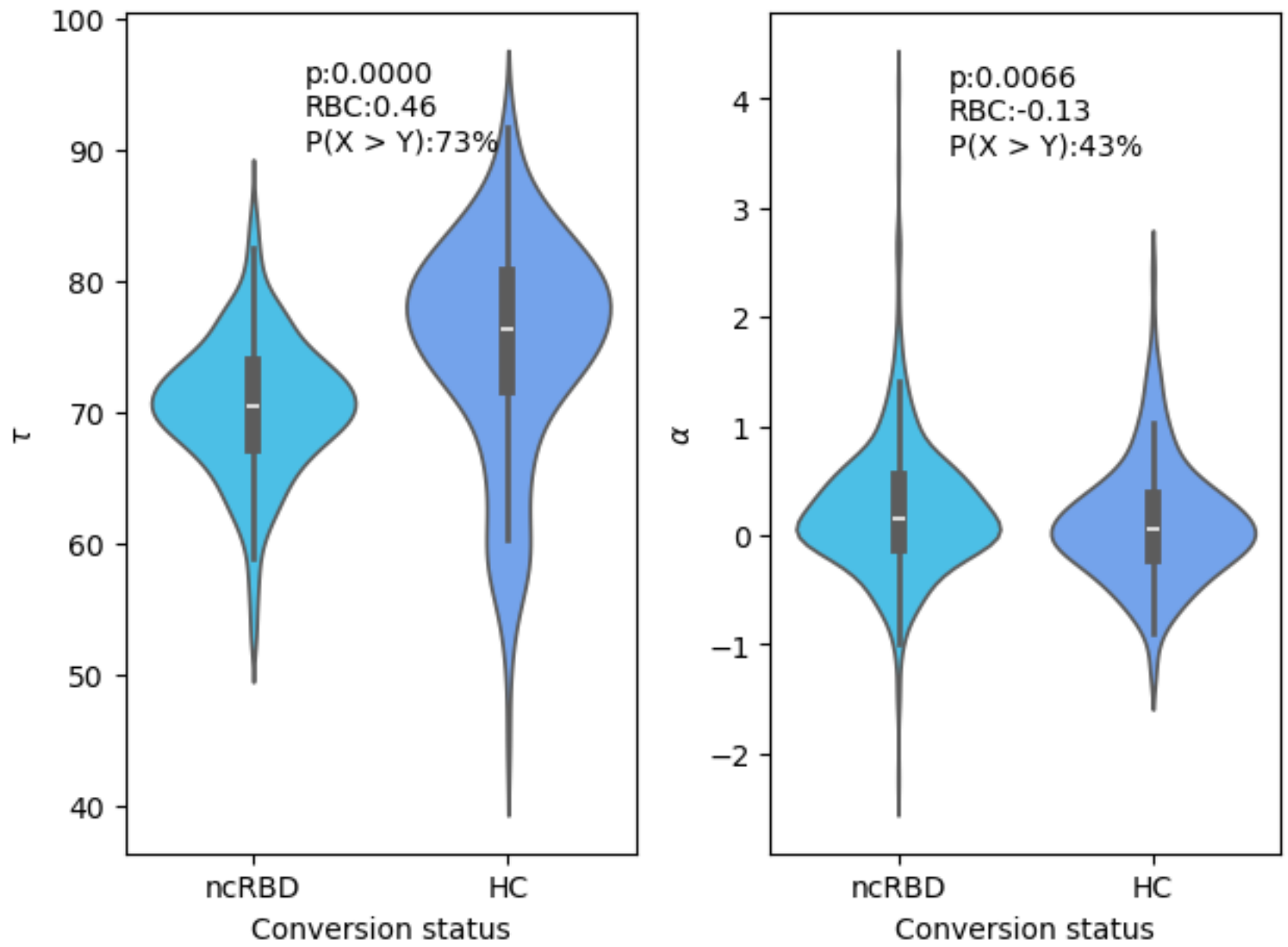

Figure S1: **Model parameters distributions between non-converters versus healthy controls.** P denotes the p-value from the Wilcoxon rank-sum test; RBD indicates the rank-biserial correlation; and P(X > Y) represents the exact probability derived from the rank-biserial correlation, corresponding to the probability that a randomly sampled value from group X exceeds one from group Y.

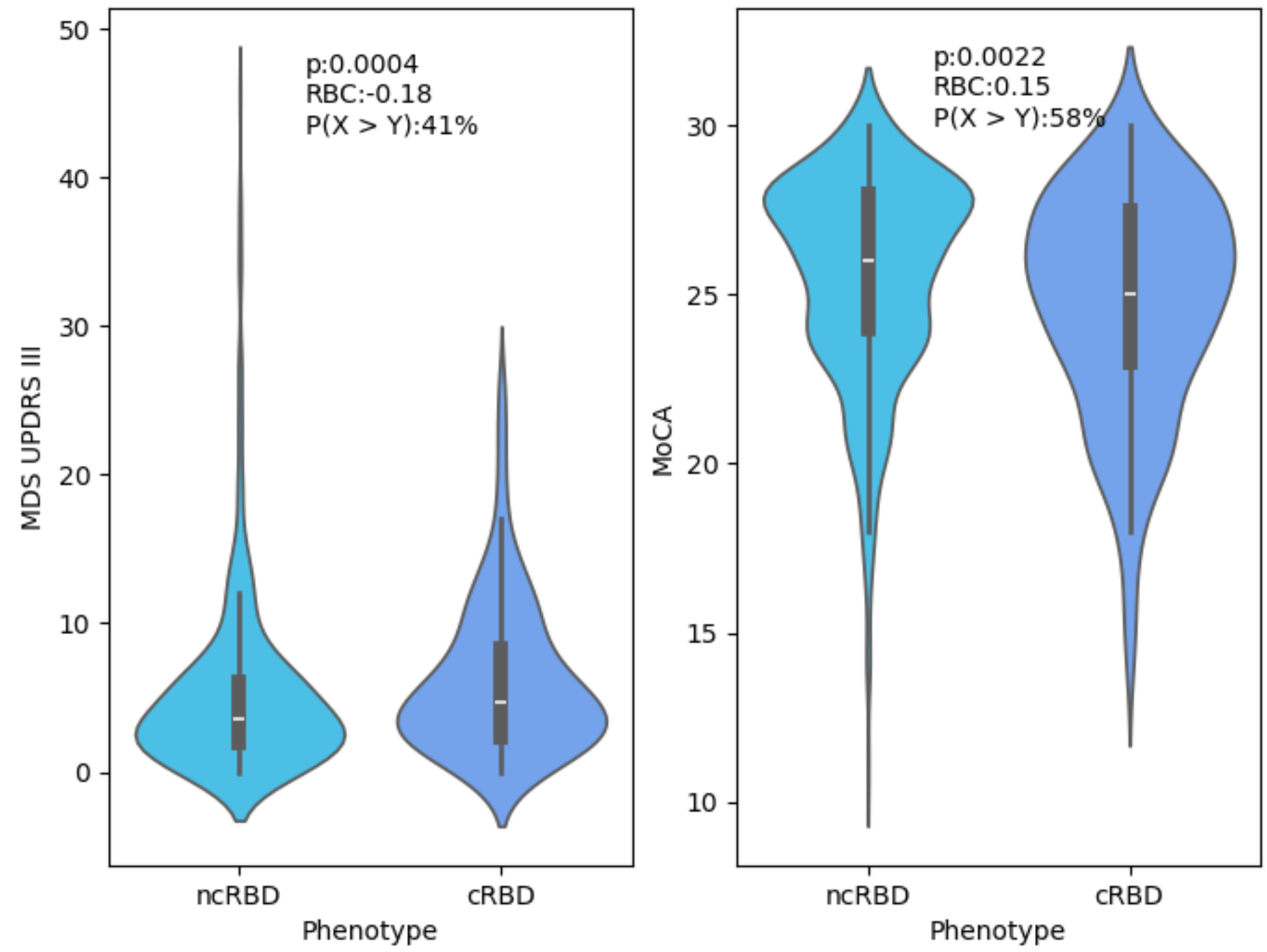

Figure S2: **Baseline MDS–UPDRS III and MoCA scores in converters versus non-converters.** P denotes the p-value from the Wilcoxon rank-sum test; RBD indicates the rank-biserial correlation; and P(X > Y) represents the exact probability derived from the rank-biserial correlation, corresponding to the probability that a randomly sampled value from group X exceeds one from group Y.

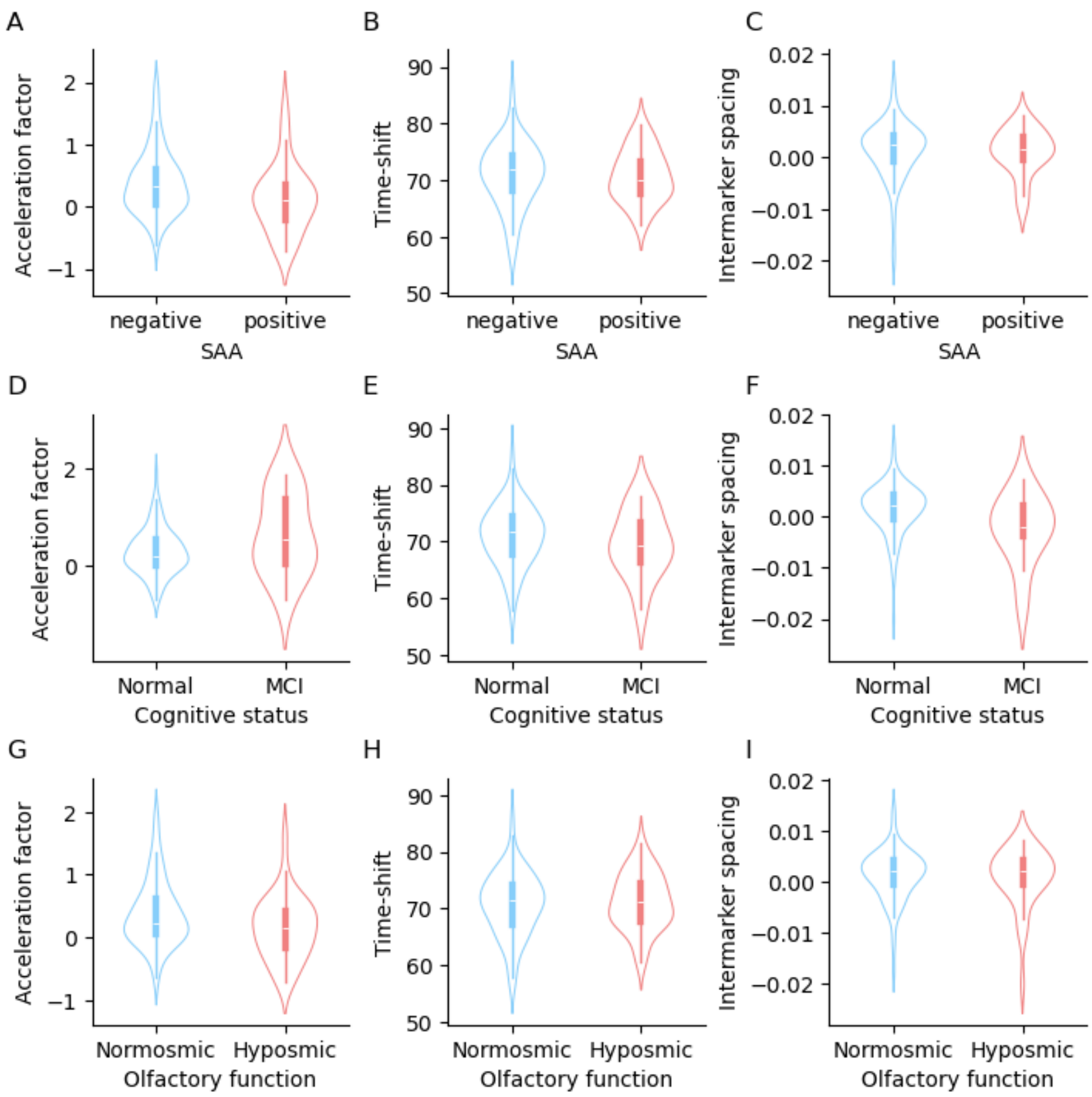

Figure S3**:** Violin plot of acceleration factor, time-shift, and intermarker spacing for a-syn biomarker (A, B, C), cognitive status (D, E, F), and hyposmia (G, H, I). Effect sizes and p-values corrected are reported in Table S3.

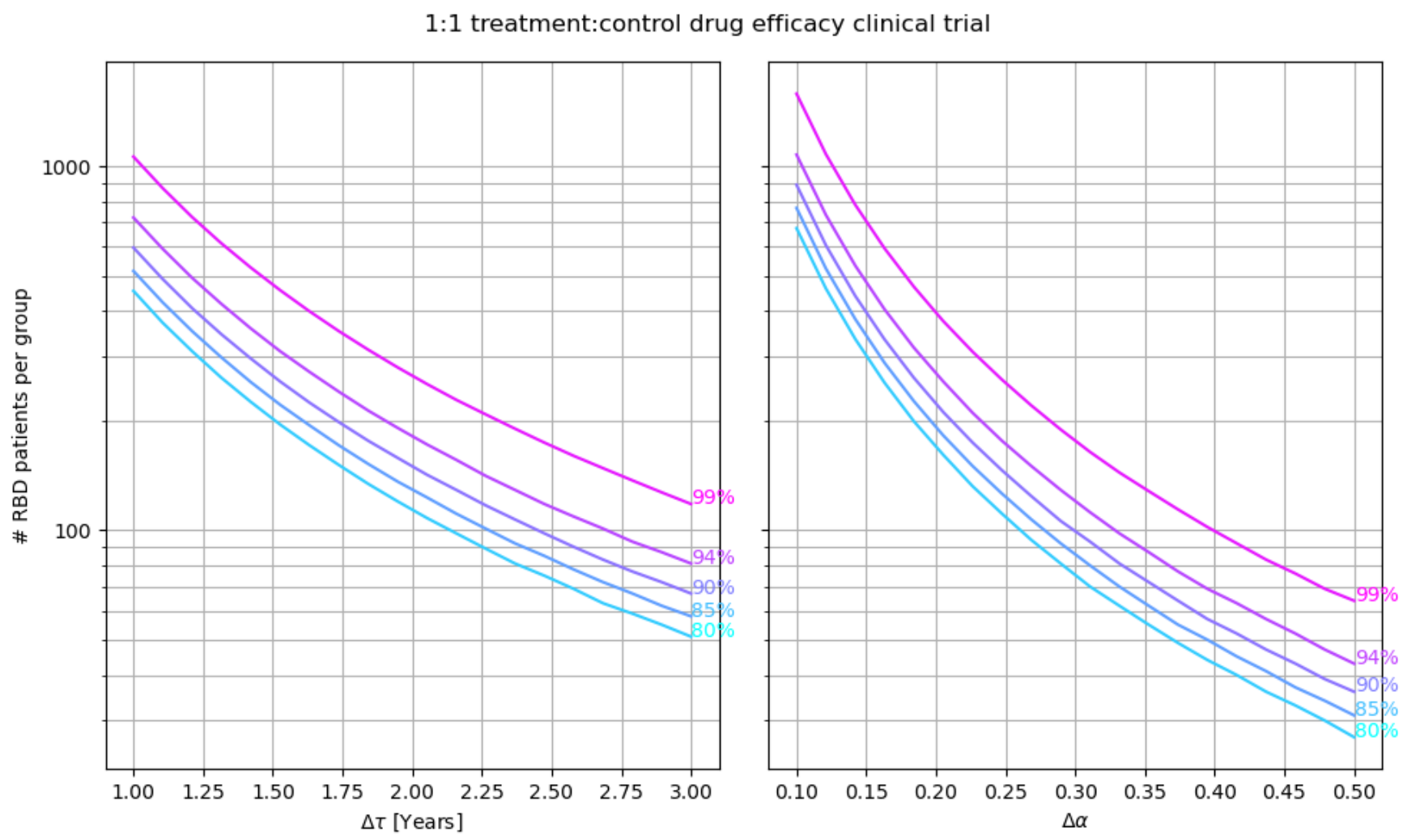

Figure S4: **Sample size calculation for a hypothetical 1:1 treatment: control drug efficacy clinical trial as a function of endpoint desired effect.** Individual lines represent sample size estimated at different desired powers ranging from 80% up to 99%.
